# Lower Protein Intake Supports Normal Growth of Full-Term Infants Fed Formula: A Randomized Controlled Trial

**DOI:** 10.3390/nu10070886

**Published:** 2018-07-10

**Authors:** Lorena G. Oropeza-Ceja, Jorge L. Rosado, Dolores Ronquillo, Olga P. García, María del C. Caamaño, Carlos García-Ugalde, Rubí Viveros-Contreras, Miguel Ángel Duarte-Vázquez

**Affiliations:** 1 Facultad de Ciencias Naturales, Universidad Autónoma de Querétaro, Campus Juriquilla, Avenida de las Ciencias s/n, Juriquilla, 76230 Querétaro, Mexico; loreniza15@gmail.com (L.G.O.-C.); jlrosado@prodigy.net.mx (J.L.R.); lolitaron@hotmail.com (D.R.); opgarciao@yahoo.com.mx (O.P.G.); mccaamano@hotmail.com (M.C.C.); 2 Centro de Investigación y Desarrollo Tecnológico en Enfermedades Crónicas (CINDETEC) A.C., Avenida Jurica 122, Parque Industrial Querétaro, 76220 Querétaro, Mexico; 3 Departamento de Investigación y Desarrollo, Nucitec, S.A. de C.V., Avenida Jurica 116, Parque Industrial Querétaro, 76220 Querétaro, Mexico; carlosragau@gmail.com; 4 Instituto de Ciencias Básicas, Universidad Veracruzana, Avenida Dr. Rafael Sánchez Altamirano s/n, Industrial las Ánimas, C.P. 91192 Veracruz, Mexico; rubiviveros@hotmail.com

**Keywords:** human milk, infant formula, infant growth

## Abstract

Infant formulas have been conventionally prepared with an excess of total protein in order to provide sufficient amounts of essential amino acids to the rapidly growing infant. However, this practice leads to higher than necessary protein intake during early infant development, inducing accelerated growth patterns correlated with the development of chronic diseases later in life. This study was aimed at assessing the safety of an infant formula enriched with bovine alpha-lactalbumin containing a total protein concentration very close to that of human milk, and determining its efficacy in the support of healthy infant growth from the first month to the fourth month of age. Healthy full-term infants ≤40 days of age were randomized in this controlled single blind trial to one of the following infant formulas: IF 1 (containing 1.0 g protein/dL; *n* = 30), IF 2 (containing 1.3 g protein/dL; *n* = 24), and IF 3 (containing 1.5 g protein/dL; *n* = 42). A control group consisting of exclusively breastfed infants (HM; *n* = 212) was included in the study. Anthropometric measurements and *Z-scores* were evaluated at baseline, at 1 month of age, and at 4 months of age. Weight gain (g/day) was similar in the IF 1 and the HM groups (*p* = 0.644), and it was significantly greater in the IF 2 and IF 3 groups than in the HM group. Growth patterns in both breastfed or IF-fed infants were in accordance with the World Health Organization (WHO) growth standards. At four months of age, the mean weight-for-age *Z-score* (WAZ) adjusted for initial value in the IF 1 group was similar to that of the HM group and significantly lower than that of the IF 2 and IF 3 groups (*p* = 0.031 and *p* = 0.014 for IF 2 and IF 3, respectively). Length-for-age (LAZ) adjusted for initial value was similar among all groups at four months of age. From 1 to 4 months of life, IF 1 containing 1.0 g protein/dL promotes growth and weight gain similar to those observed in exclusively breastfed infants. As this is a first approach to studying an IF containing total protein in a level below that recommended by international committees on nutrition, further investigations are needed to support these findings evaluating infant’s metabolic profile and growth in the long term.

## 1. Introduction

Human milk (HM) contains a unique blend of proteins that provides energy, essential amino acids, and a variety of bioactivities that support healthy growth and development during early infancy [1]. Additionally, breastfeeding may reduce the risk of chronic diseases later in life [2]. For this reason, HM is considered the gold standard of early infant nutrition and the World Health Organization (WHO) recommends that infants be exclusively breastfed for the first six months of life [3]. However, prevalence of exclusive breastfeeding in the population of infants under six months of age is only 40% worldwide [4]. Both WHO and the American Academy of Pediatrics recognize that infant formulas (IFs) are nutritionally adequate substitutes for HM when mothers cannot produce it, when breastfeeding is medically contraindicated, and when they decide not to breastfeed their babies [5].

Currently, standard IFs are based on cow’s milk proteins, which must be adjusted by the addition of whey protein concentrates to more closely resemble the whey protein-to-casein ratio of HM of approximately 65:35 [6,7]. Nevertheless, HM and IFs still differ substantially in protein composition and concentration. Mature HM provides total protein at a level of 0.9 ± 0.2 g/dL in addition to a well-balanced essential amino acid content, including tryptophan and cysteine [8]. In contrast, protein concentrations in IFs ranges from 1.26 g/dL to 2.05 g/dL in accordance with the recommendations of international guideline development committees on nutrition [9,10]. Higher protein concentrations in IFs are necessary for the delivery of sufficient quantities of essential amino acids to the rapidly growing infant because of the limited contribution of tryptophan and cysteine from cow’s milk proteins [11]. Moreover, adjustment of the whey protein-to-casein ratio in IFs results in large concentrations of beta-lactoglobulin [11], which is the predominant whey protein in cow’s milk although it is absent in HM [12,13]. Bovine beta-lactoglobulin is characterized by having the greatest proportion of branched-chain amino acids of all the cow’s milk proteins [13].

Increasing evidence indicates that higher protein intake in IF-fed infants consistently causes rapid weight gain during the first year of life [14,15]. Infants fed with IF show higher plasma concentrations of branched-chain amino acids, insulin, and insulin-like growth factor (IGF-1) compared to breastfed infants [16]. Branched-chain amino acids are known physiological stimuli of insulin secretion [17], and both insulin and IGF-1 have mitogenic and anabolic effects and stimulate adipogenesis [18]. These endocrine alterations may be the underlying mechanisms of the accelerated weight gain observed in IF-fed infants compared to breastfed infants. Rapid weight gain in early infancy has been closely associated with a greater risk of obesity and its comorbidities later in life [19,20,21,22]. Thus, lowering total protein content in IF is critical to avoid excessive exposure of infants during early development and to reduce risk of later adverse health outcomes.

Several studies have demonstrated that it is possible to reduce total protein concentration and improve whey protein quality in IFs through the addition of bovine alpha-lactalbumin isolates. Alpha-lactalbumin is a major protein in HM, accounting for 25–35% of the total protein [23]; its nutritional value relies on its excellent proportion of tryptophan and cysteine [24]. Tryptophan is the precursor to the neurotransmitter serotonin, and cysteine is a key component of glutathione and a precursor in taurine biosynthesis [12]. Additionally, peptides released following gastrointestinal digestion of alpha-lactalbumin show prebiotic and antibiotic activities and may improve the absorption of some divalent minerals [24]. Bovine alpha-lactalbumin (UniProtKB: P00711; The UniProt Consortium) shows a 75% sequence similarity to human alpha-lactalbumin (UniProtKB: P00709, NCBI (The National Center for Biotechnology Information) protein BLAST tool) in a total of 123 amino acid residues and it represents up to 17% of total protein in commercially available IFs [25]. Infants receiving IFs enriched with bovine alpha-lactalbumin containing 1.26 ± 0.02 g protein/dL at up to six months of age have shown growth outcomes similar to those of breastfed infants in weight gain, body mass index, and weight-for-age, length-for-age, and weight-for-length z scores, while demonstrating adequate gastrointestinal tolerance to IFs [14,26,27]. It is worth noting that plasma amino acid levels in IF-fed infants were found to be significantly higher than in breastfed infants, particularly the branched-chain amino acids isoleucine and valine [26]. It has also been reported that an IF with a total protein content as low as 1.15 g/dL prepared from intact cow’s milk proteins to obtain a 60:40 whey protein-to-casein ratio supports age-appropriate growth from 3 to 6 months of age [28]. Nonetheless, serum levels of urea, IGF-1, insulin, and c-peptide were elevated in IF-fed infants compared to breastfed infants [28].

Taking together the clinical evidence described above, we considered that it was possible to further reduce total protein concentration of standard IF by increasing alpha-lactalbumin content up to a level close to that of mature HM. The aim of this study was to evaluate the safety of an experimental IF enriched with bovine alpha-lactalbumin containing 1.0 g protein/dL (IF 1) by identifying adverse effects, and to investigate its efficacy in the support of suitable growth in healthy full-term infants during the first four months of life. Growth outcomes from IF 1-fed infants were compared to those of exclusively breastfed infants.

## 2. Subjects and Methods

### 2.1. Study Design

The study was designed to determine whether an experimental IF with a total protein content of 1.0 g/dL enriched with bovine alpha-lactalbumin (26% of total protein, IF 1) leads to slower weight gain in healthy full-term infants from one to four months of age compared to: (i) an IF with a total protein concentration of 1.3 g/dL also enriched with bovine alpha-lactalbumin (26% of total protein, IF 2); and (ii) a standard IF with a total protein content of 1.5 g/dL (IF 3). It was hypothesized that IF 1 would produce a weight gain and a growth pattern similar to those observed in breastfed infants. The hypothesis was tested in a single-blind controlled trial in which infants ≤40 days of age were randomly assigned to one of the IFs under study from 1 month to 4 months of life. A control group (HM group) of breastfed infants was included in the study, as recommended by the European Society for Paediatric Gastroenterology, Hepatology and Nutrition (ESPGHAN) and the U.S. Food and Nutrition Board of the Institute of Medicine of the National Academies [14]. CONSORT 2010 checklist (supplementary) and flow diagram were completed for reporting the results of this randomized trial.

### 2.2. Study Size and Power Calculation

In the present study, sample size was calculated to have sufficient power (80%) to detect a difference in weight gain of 5 g/day among the IF groups from baseline to four months of age [15,26], with an estimated standard deviation of 5.5 g/day and a type I error (α) of 5%. With these considerations, 18 infants were necessary per group. Also, assuming an estimated loss-to-follow-up rate of 25%, there was a need to enroll 23 infants per each intervention group.

### 2.3. Study Population and Ethics

Infants eligible for study participation met the following inclusion criteria: (a) healthy full-term singleton newborns (≥37 weeks’ gestation); (b) birth weight ≥2500 g and ≤4000 g; (c) Second Apgar score >8; (d) age of up to 40 days when entering the study. Infants with congenital heart defects, congenital illnesses or malformations, severe gastrointestinal, kidney, liver, or central nervous system diseases or metabolic disorders, or those born to mothers with gestational diabetes mellitus were excluded from the study. Infants were recruited from six medical centers of the National Ministry of Health located in Queretaro, Mexico.

Of the 320 infants screened, 308 met the inclusion criteria and were enrolled in the study, which was conducted from February 2016 to November 2017. Parents were approached and informed about the procedures and objectives, and then they were invited to participate in the study if the exclusion criteria did not apply. If mothers could not or intended not to breastfeed their babies, participation in the intervention groups was offered, with provision of IFs at no cost. If consent was obtained, infants were randomly assigned to any of the three study IFs. If the mother decided to exclusively breastfeed for 4 months, her infant was included in the control group of exclusively breastfed infants. Type of feeding of infants participating in the present study (exclusively IF, exclusively breastfed, or mixed feeding) before enrollment was also recorded. For the intervention groups, noncompliance was defined as infants receiving any mixed feeding (breastfeeding plus study IF or non-study IF) and/or any complementary food before four months of age. The participants’ mothers were trained to maintain a diary on frequency of gastrointestinal (GI) events such as vomiting, constipation, diarrhea, abdominal inflammation, and crying. This information was collected by study personnel at each study visit. Parents also recorded feeding guideline noncompliance in this diary.

The study was approved by the Bioethics Committee of the Universidad Autónoma de Querétaro (UAQ, Queretaro, Mexico) and it was conducted in accordance with the guidelines of the Declaration of Helsinki and of the good clinical practice guidelines of the International Conference on Harmonisation. Ethics committees from each medical center also authorized the study protocol. Written informed parental consent was obtained for each infant. The study was registered at ClinicalTrials.gov (NCT03513991).

### 2.4. Nutritional Composition of Study Formulas

The IF-fed infants were randomized to one of the study IFs using a computerized automatic randomization system [29]. IFs were manufactured in powder form by Nucitec S.A. de C.V. (Queretaro, Mexico) and were provided to the participants’ parents at no cost. The nutritional compositions of all IFs under study were appropriate for healthy full-term infants during the first six months of life, according to recommendations of ESPGHAN and the Codex Alimentarius Commission [9,30]. Energy and protein concentration and composition are detailed in Table 1. IF 1 and IF 2 were enriched with bovine alpha-lactalbumin using the whey protein isolate Hilmar 8800 (Hilmar Cheese Company, Hilmar, California, USA). These two IFs were also prepared using cow’s milk with beta-casein (BC) A2 free of BC A1 from genetically selected animals [31] (Ecológico Tierra Viva, Dolores Hidalgo, Mexico). IF 3 was prepared from native cow’s milk. The three formulas were packaged in the same blue presentation and labeled with the nutritional information, powder reconstitution instructions, and dosing recommendations. Each formula was identified by a batch number that did not identify the parents of the infants.

### 2.5. Study Visits and Anthropometric Measures

The IFs were distributed with uniform instructions for powder preparation. Three packages of study IF (400 g each) were duly delivered to the mothers every week at each medical center. The supply of study IF was sufficient to cover infant feeding for 7 days. Feeding compliance was assessed by asking the mothers to return the empty packages of IF at the following visit.

Birth weight and length were obtained from hospital data. Anthropometric measurements (infant weight, length, and head circumference) were obtained at visits to the medical center: at study entry (baseline) and at one and four months of age. Weight was determined with a digital scale (Seca Mod 334, Hamburg, Germany) equipped with a measuring rod (Seca Mod 232, Hamburg, Germany). Head circumference was measured with an ergonomic circumference measuring tape (Seca Mod 201, Hamburg, Germany). All measurements were taken in duplicate with an accuracy of 10 g for weight and 0.1 cm for length and head circumference. Demographic information was collected at baseline using a validated questionnaire [34].

### 2.6. Data Processing and Statistical Analysis

Analysis of demographic data from infants participating in the present study was performed by a Pearson chi-square statistical test. Growth velocity, including gain in weight, length, and head circumference was calculated by dividing results of anthropometric measures by the exact number of days between visits of each subject. Weight gain is expressed as mean g/day (95% of Confidence Interval (CI)) whereas gain in length and head circumference are reported as mean cm/month ± Standard Deviation (SD). weight-for-age *Z-score* (WAZ), length-for-age (LAZ), weight-for-length *Z-score* (WLZ), head circumference-for-age *Z-score* (HCAZ), and body mass index-for-age *Z-score* (BMIAZ) were calculated using the WHO Multicenter Growth Reference 2006 software [35]. Descriptive analyses were performed for all variables. Post-intervention means of weight, length, and head circumference *Z-scores* were compared among groups with analysis of variance (ANOVA) adjusted for baseline values. Growth velocity was also adjusted by gender. The Fisher’s least significant difference (LSD) post hoc test was used for multiple comparisons between groups. All statistical analyses were performed using IBM (International Business Machines, Armonk, NY, USA) Statistical Package SPSS 22.0. A *p*-value < 0.05 was considered significant.

## 3. Results

### 3.1. Subjects Characteristics

**Figure 1:**
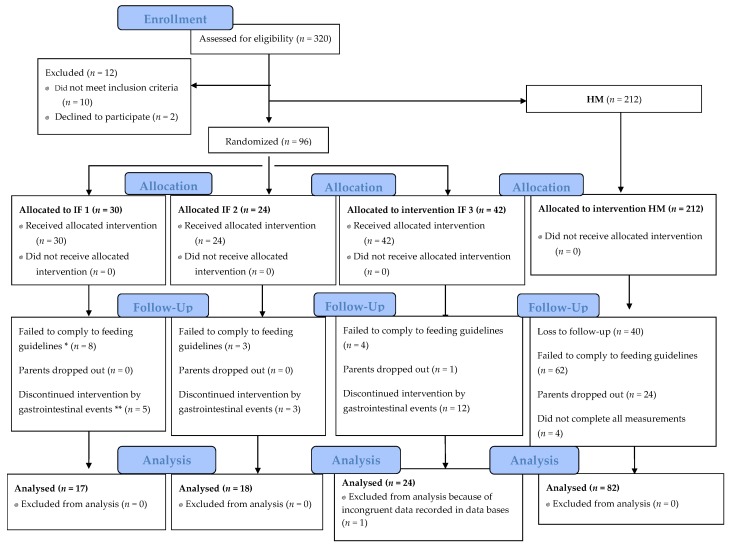
Study flowchart. * Failed to comply to feeding guidelines refers to infants who received mixed feeding (breastfeeding and infant formula) and/or complementary food before four months of age. ** Mothers reported gastrointestinal events such as vomiting, constipation, diarrhea, abdominal inflammation, and crying.

Three hundred eight infants were enrolled in the study, of whom 212 made up the control group of exclusively breastfed infants. A total of 96 infants were randomized to receive one of the study formulas (Figure 1). A total of 166 infants were voluntarily withdrawn during the study, representing 53% of the total included population. Of the dropout population, 130 were from the control group and dropout was mainly due to noncompliance with feeding guidelines. However, this did not affect statistical power in the growth parameters.

It was observed that the main reason for withdrawal in the infant formula groups was GI events. The IF 3 group had more GI event-related withdrawals than IF 1 and IF 2 (8.3%, 16.7%, and 28.6%, respectively). There were no withdrawals due to gastrointestinal events in the HM group.

Type of feeding from birth to enrollment by group is shown in Table 2. In the IF groups, infants were predominantly fed with a commercially available IF before enrollment.

Table 3 shows the baseline characteristics of the infants and mothers who completed the study. Weight at birth in IF 2 was significantly lower than in HM and IF 1 (*p* = 0.001) (Table 2). No significant differences were found in other infant characteristics, delivery type, or sex (F = 2.358, gl = 3, *p* = 0.074).

### 3.2. Growth Velocity

ANOVA adjusted by gender showed significant differences among groups in weight gain (Table 4). Weight gain after intervention was not significantly different between the IF 1 and HM groups (*p* = 0.646), but weight gain in these two groups was significantly lower than in the IF 2 and IF 3 groups (*p* = 0.007 and *p* = 0.001, respectively). There were no significant differences in mean length gain and mean head circumference gain among groups (*p* > 0.05).

### 3.3. Anthropometric Measurements and Growth (Z-scores)

At baseline, there were significant differences in weight, as revealed by one-way ANOVA (F = 2.8, df = 3, *p* = 0.042), as well as in head circumference (F = 3.47, df = 3, *p* = 0.018) (Table 5). Post hoc analysis showed that baseline weight in the IF 2 group was significantly lower than in the IF 3 (*p* = 0.030) and HM (*p* = 0.005) groups (Table 4). Head circumference in the IF 2 group was significantly lower than in the IF 1 (*p* = 0.032), IF 3 (*p* = 0.016), and HM (*p* = 0.002) groups (Table 5). There were no significant differences in length (F = 2.3, df = 3, *p* = 0.082). These initial values were used as covariates and did not affect results after four months.

At baseline, there were significant differences in WAZ, LAZ, and HCAZ, as revealed by one-way ANOVA (F = 4.77, df = 3, *p* = 0.003; F = 3.57, df = 3, *p* = 0.016; F = 6.04, df = 3, *p* = 0.001; respectively) (Table 6). Post hoc analysis showed that WAZ in the IF 2 group was significantly lower than in the IF 3 (*p* = 0.030) and HM (0.005) groups. Head circumference in the IF 2 group was significantly lower than in the IF 1 (*p* = 0.032), IF 3 (*p* = 0.016), and HM (*p* = 0.002) groups. There were no significant differences in WLZ (F = 0.85, df = 3, *p* = 0.468) or BMIZ (F = 1.9, df = 3, *p* = 0.122) among the groups.

At four months of age, after adjusting for initial values, there were significant differences in WAZ (F = 2.7, df = 3, *p* = 0.049). Post hoc analysis showed that WAZ in IF 1 was significantly lower than in the IF 2 (*p* = 0.031) and IF 3 (*p* = 0.014) groups and was similar to that of the HM group (*p* = 0.165). There were no significant differences in WLZ (F = 1.8, df = 3, *p* = 0.139), LAZ (F = 1.2, df = 3, *p* = 0.302), HCAZ (F = 1.6, df = 3, *p* = 0.184), or BIAZ (F = 2.1, df = 3, *p* = 0.107) among the groups.

## 4. Discussion

Our results show normal growth with 1.0 g of protein per 100 mL intake through infant formula. We found that healthy full-term infants fed with a new lower-protein formula at four months of age had a weight gain and a WAZ (25.8 g/day; −0.6 *Z-score*) similar to those of the breastfed infants (27.0 g/day; −0.4 *Z-score*). Although the number of infants completing the study in groups IF 1 and IF 2 were the minimum required, the statistical power remained above 80% (β = 0.890). Our results are consistent with those of Fleddermann et al. [15] and Trabulsi et al. [26], who reported a weight gain of 26.7 ± 6.4 g/day and 26.6 ± 5.4, respectively, in breastfed infants at four months of age.

It has been demonstrated that infant formulas with a protein content ranging from 1.2 g protein/dL (1.8 g/100 kcal) [27,32,33,36] to 1.5 g protein/dL (2.25 g/100 kcal) [21] support healthy early growth comparable to the WHO growth standards. This study found that three different protein compositions promoted growth *Z-scores* within the normal WHO ranges for infant growth from 1 month to 4 months of life. However, it is notable that the higher protein concentration of IF 2 and IF 3 produced rapid weight gain during this period. Our data are consistent with another controlled clinical trial [15] that observed a weight gain of 30.2 ± 6.3 g/day with 1.28 g protein/dL. Recently, another study showed weight gains of 32.5 ± 6.1 and 32.8 ± 6.8 with 1.2 and 1.7 g protein/dL, respectively [37].

The concentration of 1.2 g protein/dL has been considered a low infant formula protein content, but it is clear that this amount of protein promotes rapid weight gain [14,15,26,27]. The rapid weight gain seen in the IF 2 group was most likely due to the fact that these infants started with a lower weight, WAZ, and LAZ than the other three groups. The amount of protein in IF 2 is safe and appropriate for infants with slightly low weight within the WHO ranges. The infant formula containing 1.0 g of protein/dL had the effect of reducing rapid weight gain between baseline and four months of age.

The amount of protein in IFs plays an important role in early programming and it is causative for a more rapid weight gain [21]. Rapid weight gain during infancy has been shown to be a risk factor for obesity and overweight in early childhood, adolescence, and even adulthood [14,19,22,38,39]. The currently established mechanism is overactivation of the infant’s mTORC1 (mechanistic target of rapamycin complex 1) signaling pathways, which results in high plasma insulin [40], IGF-1 [28], branched-chain amino acids (insulinogenic) [41], and urinary C-peptide concentrations [42,43] compared to breastfed infants. When IGF-1 and insulin concentrations increase as a result of IF, exaggerated mTORC1 signaling is overexpressed [44]. This increase of mTORC1 not only accelerates infant growth but may also lead to the development of allergies and to metabolic and immunological programming [44]. This mechanism is thought to support the “early protein hypothesis”.

It is also important to consider the implications of high protein consumption during infancy. High protein intake, regardless of caloric content, affects kidney function and size in healthy infants [45]. Escribano et al. demonstrated a direct effect of protein intake on body growth parameters and kidney size [45]. Infants who receive infant formula with high protein show high renal workloads, blood urea [28,36,45], urine osmolarity, creatinine, and kidney size [45] compared to infants fed low protein content formula and human milk. Thus, much effort has been made to improve protein quality and reduce protein quantity in IFs. One approach consists of increasing the proportion of alpha-lactalbumin to levels similar to those found in human milk. The very-low-protein infant formula evaluated here was enriched with alpha-lactalbumin at a proportion of 26% alpha-lactalbumin of total protein. Alpha-lactalbumin is the most abundant protein in breast milk, where it accounts for 25–30% of total protein. It has a good balance of essential amino acids, mainly tryptophan and cysteine, which are unbalanced in cow’s milk protein and therefore in infant formula. Currently, there are commercially available bovine alpha-lactalbumin isolates, which when added to infant formulas increase the content of amino acids tryptophan and cysteine; this improves the quality of the protein and consequently the protein quantity can be reduced.

This study provides additional evidence considering the withdrawal rate of subjects who were fed with infant formula. The number of drop outs due to gastrointestinal events (vomiting, constipation, diarrhea, abdominal inflammation, and crying) was significantly lower in IF 1 and IF 2 groups than in the IF 3 group (*p* = 0.001), possibly because both formulas contained BC A2. In a pilot study, BC A2 was better tolerated than BC A1 by humans [46]. Additionally, IF 1 and IF 2 contained a higher proportion of alpha-lactalbumin than IF 3. The improved GI profile observed in the present study may be attributed to a formula matrix that more closely resembles HM. Alpha-lactalbumin is a well-tolerated and highly digestible protein, which may have contributed to a lower rate of dropout due to gastrointestinal adverse events in the formulas enriched with alpha-lactalbumin. Lien et al. [47] and Davis [41] found improved tolerance in infants fed with alpha-lactalbumin-enriched formula, as demonstrated by superior acceptability and tolerance ratings in experimental formula versus standard formula. Feeding noncompliance was common in all groups for two primary reasons: (1) the mother’s belief that mixed feeding (breastfeeding and infant formula) is needed to satisfy the infant’s hunger, and (2) the mother’s report of feeling that the infant needs to taste food to satisfy his/her hunger.

Limitations of this study were the time of intervention and measurements of body composition at 1 and 4 months of age. We cannot know how growth would be affected in infants fed IF 1 in the long term. This study was also unable to measure metabolic profile data for parameters such as blood urea nitrogen (BUN), blood amino acids, insulin, IGF-1, and c-peptide levels.

## 5. Conclusions

This is the first study that demonstrates that an infant formula with 1.0 g protein/dL enriched with bovine alpha-lactalbumin at a level similar to that of human milk (26%) containing A2 Beta-Casein supports normal growth in healthy full-term infants from 1 month to 4 months of life. Experimental infant formula with very low protein (i.e., a lower protein concentration than recommended by ESPGHAN and Codex Alimentarius) slows weight gain from 1 to 4 months of age, which is considered a risk factor for later development of obesity. Although this is a first approach, our results suggest that enrichment of IFs with bovine alpha-lactalbumin allows the amount of total protein to be decreased as low as 1.0 g/dL, similar to mature human milk, which would result in a lower metabolic workload for infants. However, we recommend further investigations to support these findings, and to evaluate endocrine and plasma amino acids profiles in infants fed experimental IF. It also will be necessary to evaluate the effects of this IF on infant growth in the long term.

## Figures and Tables

**Table 1:** Energy and protein profiles of the three study infant formulas and human milk.

	Infant Formulas			HM **
	IF 1 *	IF 2 *	IF 3 *	
Energy (kcal/dL)	65	68	70	63.6 ± 4.5
Protein profile				
Whey:Casein ratio	65:35	65:35	60:40	70:30
Protein content (g/dL) ^a^	1.0	1.3	1.5	0.9 ± 0.2
Protein (g/100 kcal)	1.43	1.9	2.18	1.28
Beta-casein variant ^b^	BCA2	BCA2	BCA1/BCA2	Human beta-casein
Alpha-lactalbumin (%)	26	26	11	33

IF: infant formula; HM: human milk. * Protein profile analyzed previously [32]; ^a^ Protein content calculated according to manufacturer’s instructions; ** Values of protein profile in human milk as reported in the literature [7,8,33]. ^b^ BCA2: A2 beta-casein variant; BCA1: A1 beta-casein variant.

**Table 2:** Feeding of infants from birth to enrollment by group, as reported by mothers.

	IF 1		IF 2		IF 3			HM	
	*n* = 17		*n* = 18		*n* = 24			*n* = 82	
Exclusively IF	3	17.6%	5	27.8%	6	25.0%		1	1.2%
Exclusively Breastfed	6	35.3%	2	11.1%	7	29.2%		67	81.7%
Mixed feeding	8	47.1%	11	61.1%	11	45.8%		14	17.1%
Data are shown as: *n* (%)									

**Table 3:** Baseline characteristics of the participants who completed the study.

	Feeding Group				*p*
	IF 1	IF 2	IF 3	HM	
	*n* = 17	*n* = 18	*n* = 24	*n* = 82	
**Infant Characteristics**					
Age at baseline (d)	19 ± 11	21 ± 12	22 ± 11	21 ± 12	0.803
Weight at birth (g)	3116 ± 250	2789± 391 ^a^	2999 ± 399	3131 ± 380	0.004
Length at birth (cm)	50.0 ± 1.6	49.1 ±2.7	49.2 ± 2.1	49.9 ± 2.1	0.349
Gestational age (wk)	39.1 ±0.9	38.3 ± 1.7	38.6 ± 1.3	39.0 ± 1.1	0.112
**Delivery**					
Caesarean	6 (35%)	7 (39%)	11 (46%)	36 (45%)	0.981
Forceps	1 (7%)	0 (0)	1 (4%)	1 (1%)	
Normal vaginal	10 (58%)	12 (61%)	12 (50%)	45 (54%)	
**Sex**					
Male	8 (47%)	12 (61%)	10 (42%)	44 (54%)	0.467
Female	9 (53%)	7 (39%)	14 (58%)	38 (46%)	
**Mother’s Characteristics**					
Age at baseline (y)	21.1 ± 4.2	26.6 ± 7.8	24.9 ± 5.2	25 ± 5.4	0.074
Mother’s education (y)	8.0 ± 2.6	7.7 ± 3.3	8.9 ± 3.0	8.9 ± 2.2	0.211
Crowding (people per room)	3.0 ± 1.6	2.5 ± 0.8 ^b^	3.0± 1.6	3.6 ± 1.8	0.041

Values are presented as means ± Standard Deviation (SD). IF 1: experimental IF containing 1.0 g protein/dL enriched with bovine alpha-lactalbumin (26% of total protein); IF 2: IF containing 1.3 g protein/dL also enriched with bovine alpha-lactalbumin (26% of total protein); IF 3: standard IF containing 1.5 g protein/dL; HM: human milk group; ^a^ Significantly different from IF 1 (*p* = 0.006) and HM (*p* = 0.001); ^b^ Significantly different from HM (*p* = 0.015).

**Table 4:** Gains in weight, length, and head circumference of infants by feeding group from 1 to 4 months of age.

Growth Velocity	Feeding Group				
	IF 1	IF 2	IF 3	HM	*p*
	*n* = 17 95% CI	*n* = 18 95% CI	*n* = 24 95% CI	*n* = 82 95% CI	
Weight gain (g/day)	25.8 (21.9, 29.2) ^a^	32.3 (28.9, 35.8)	31.5 (28.5, 34.5)	27.0 (25.5, 28.7) ^b^	0.001
Length gain (cm/mo)	2.4 (2.1, 2.7)	2.6 (2.3, 2.9)	2.6 (2.3, 2.8)	2.4 (2.3, 2.6)	0.646
Head circumference gain (cm/mo)	1.2 (1.1, 1.4)	1.3 (1.2, 1.5)	1.3 (1.0, 1.4)	1.2 (1.1, 1.3)	0.530

CI: Confidence Interval. IF 1: experimental IF containing 1.0 g protein/dL enriched with bovine alpha-lactalbumin (26% of total protein); IF 2: IF containing 1.3 g protein/dL also enriched with bovine alpha-lactalbumin (26% of total protein); IF 3: standard IF containing 1.5 g protein/dL; HM: human milk group. Data are shown as mean ± SD; ^a^ IF 1 group was significantly different from the IF 2 (*p* = 0.016) and IF 3 (*p* = 0.006) groups; ^b^ HM group was significantly different from the IF 2 (*p* = 0.007) and IF 3 (*p* = 0.001) groups.

**Table 5:** Weight, length, and head circumference measurements at baseline, at 4 months of age, and adjusted change.

	Feeding Group								*p*
	IF 1		IF 2		IF 3		HM		
	*n* = 17	95% CI	*n* = 18	95% CI	*n* = 24	95% CI	*n* = 82	95% CI	
**Weight (g)**									
Baseline	3617.6	(3333.7, 3877.4)	3276.6 ^a^	(3003.5, 3549.8)	3679.1	(3310.0, 3911.2)	3718.7	(3587.4, 3850.1)	0.042
4 mos. of age *	6330.7	(6011.0, 6650.4)	6482.8	(6174.7, 6791.0)	6674.1	(6413.0, 6935.1)	6470.4	(6328.3, 6612.5)	0.396
Adjusted change	2697.4	(2738.8, 3015.1)	2856.4	(2549.0, 3163.8)	2997.4	(2737.5, 3257.2)	2834.1	(2692.9, 2975.4)	0.531
**Length (cm)**									
Baseline	50.1	(48.9, 51.2)	48.8	(47.7, 49.9)	50.3	(48.8, 51.3)	50.5	(50.0, 51.0)	0.082
4 mos. of age *	59.9	(58.9, 60.7)	59.9	(59.0, 60.7)	60.6	(59.9, 61.3)	60.4	(60.0, 60.8)	0.365
Adjusted change	9.7	(8.8, 10.6)	9.8	(8.9, 10.6)	10.4	(9.6, 11.1)	10.3	(9.9, 10.7)	0.472
**Head Circumference (cm)**									
Baseline	35.6	(34.9, 36.4)	34.5 ^b^	(33.6, 35.3)	35.7	(34.9, 36.5)	35.8	(35.5, 36.2)	0.018
4 mos. of age *	40.2	(39.7, 40.7)	40.6	(40.1, 41.1)	40.9	(40.4, 41.3)	40.8	(40.5, 41.0)	0.180
Adjusted change	4.9	(4.5, 5.4)	4.8	(4.4, 5.3)	5.2	(4.8, 5.6)	5.2	(4.9, 5.3)	0.494

CI: Confidence Interval. IF 1: experimental IF containing 1.0 g protein/dL enriched with bovine alpha-lactalbumin (26% of total protein); IF 2: IF containing 1.3 g protein/dL also enriched with bovine alpha-lactalbumin (26% of total protein); IF 3: standard IF containing 1.5 g protein/dL; HM: human milk group. CI: Confidence Interval. * Changes from baseline to 4 months of age and means were adjusted by initial value (ANCOVA); ^a^ IF 2 group was significantly different from IF 3 (*p* = 0.030) and HM (*p* = 0.005); ^b^ IF 2 group was significantly different from IF 1 (*p* = 0.032), IF 3 (*p* = 0.016), and HM (*p* = 0.002).

**Table 6:** Mean *Z-score* values (95% CI) at baseline, 1 month of age, and 4 months of age by feeding group.

	Feeding Group						HM		*p*
	IF 1		IF 2		IF 3				
	*n* = 17	95% CI	*n* = 18	95% CI	*n* = 24	95% CI	*n* = 82	95% CI	
**WAZ**									
Baseline	−0.5	(−1.0, −0.1)	−1.3 ^a^	(−1.7, −0.9)	−0.9	(−1.2, −0.5)	−0.5	(−0.7, −0.3)	0.003
1 mo.	−0.8	(−0.9, −0.3)	−0.7	(−0.8, −0.5)	−0.5	(−0.7, −0.4)	−0.6	(−0.7, −0.5)	0.065
4 mos.	−0.6	(−1.0, −0.3)	−0.1 ^b^	(−0.4, 0.3)	0.0	(−0.4, 0.3)	−0.4	(−0.5, −0.2)	0.049
**WLZ**									
Baseline	0.7	(0.18, 1.2)	0.2	(−0.3, 0.7)	0.5	(0.0, 0.9)	0.6	(0.4, 0.8)	0.468
1 mo.	1.0	(0.6, 1.3)	0.9	(0.5, 1.2)	0.9	(0.6, 1.2)	1.0	(0.8, 1.1)	0.931
4 mos.	0.7	(0.3, 1.1)	1.1	(0.8, 1.5)	1.0	(0.7, 1.3)	0.7	(0.5, 0.9)	0.139
**LAZ**									
Baseline	−1.3	(−1.8, −0.8)	−2 ^a^	(−1.8, −0.8)	−1.6	(−2.1, −1.2)	−1.2	(−1.4, −0.9)	0.016
1 mo.	−1.6	(−1.8, −1.4)	−1.5	(−1.7, −1.3)	−1.4	(−1.6, −1.4)	−1.5	(−1.6, −1.4)	0.361
4 mos.	−1.5	(−1.9, −1.1)	−1.1	(−1.5, −0.7)	−1	(−1.4, −0.6)	−1.2	(−1.4, −1.0)	0.302
**HCAZ**									
Baseline	−0.2	(−0.7, 0.2)	−1.3 ^c^	(−1.8, −0.8)	−0.5	(−0.9, −0.1)	−0.2	(−0.4, −0.0)	0.001
1 mo.	−0.5	(−0.7, −0.3)	−0.4	(−0.6, −0.2)	−0.4	(−0.5, −0.2)	−0.4	(−0.5, −0.3)	0.578
4 mos.	−0.5	(−0.9, −0.2)	−0.2	(−0.5, 0.1)	−0.1	(−0.4, 0.1)	−0.3	(−0.5, −0.2)	0.184
**BMIZ**									
Baseline	0.2	(−0.2, 0.7)	−0.3	(−0.8, 0.0)	0	(−0.4, 0.4)	0.2	(0.0, 0.4)	0.122
1 mo.	0.1	(−0.1, 0.4)	−0.2	(−0.1, 0.3)	0.3	(0.1, 0.5)	0.3	(0.2, 0.4)	0.337
4 mos.	0.3	(−0.1, 0.7)	0.8	(0.4, 1.2)	0.7	(0.4, 1.1)	0.4	(0.3, 0.6)	0.107

CI: Confidence Interval. IF 1: experimental IF containing 1.0 g protein/dL enriched with bovine alpha-lactalbumin (26% of total protein); IF 2: IF containing 1.3 g protein/dL also enriched with bovine alpha-lactalbumin (26% of total protein); IF 3: standard IF containing 1.5 g protein/dL; HM: human milk group. Means were adjusted for initial value, except at baseline; ^a^ IF 2 was significantly different from IF 1 (*p* = 0.009) and HM (*p* = 0.001); ^b^ IF 1 was significantly different from IF 2 (*p* = 0.031) and IF 3 (*p* = 0.014); ^c^ IF 2 was significantly different from IF 1 (*p* = 0.002), IF 2 (*p* = 0.010), and HM (*p* = 0.001).

## Supplementary Materials

The following are available online at http://www.mdpi.com/2072-6643/10/7/886/s1, Table S1: CONSORT 2010 checklist of information to include when reporting a randomised trial *.
